# Adversarial Sample Generation Method Based on Frequency Domain Transformation and Channel Awareness

**DOI:** 10.3390/s25123779

**Published:** 2025-06-17

**Authors:** Yalin Gao, Dongwei Xu, Huiyan Zhu, Qi Xuan

**Affiliations:** Institute of Cyberspace Security, College of Information Engineering, Zhejiang University of Technology, Hangzhou 310023, China; gaoyalin97@163.com (Y.G.); xuanqi@zjut.edu.cn (Q.X.)

**Keywords:** channel estimation, deep learning, frequency-domain transformation, adversarial attack, wireless communication system

## Abstract

In OFDM wireless communication systems, low-resolution channel characteristics and noise interference pose significant challenges to accurate channel estimation. To solve these problems, we propose a super-resolution denoising residual network (SDRNet), which combines the advantages of the super-resolution convolutional neural network (SRCNN) and the denoising convolutional neural network (DnCNN) to construct a pilot-based OFDM signal model, train SDRNet using OFDM pilot data containing Gaussian noise, and optimize its feature enhancement ability in frequency-selective fading channels. To further explore the role of channel estimation in communication security, we propose a frequency-domain adversarial attack method based on SDRNet output. This method first converts the time-domain signal to the frequency domain by using the Fourier transform and then applies Gaussian noise and selective masking. By integrating the channel gradient information, the adversarial perturbation we generated significantly improves the attack success rate compared with the non-channel awareness method. The experimental results show that SDRNet is superior to traditional algorithms (such as the least square method, minimum mean square error estimation, etc.) in both mean square error and bit error rate. Furthermore, the adversarial samples optimized through channel awareness frequency-domain masking exhibit stronger attack performance, confirming that accurate channel estimation can not only enhance communication reliability but also provide key guidance for adversarial perturbation. The experimental results show that under the same noise conditions, the MSE of SDRNet is significantly lower than that of LS and MMSE. The bit error rate is lower than 0.01 when the signal-to-noise ratio is 10 dB, which is significantly better than the traditional algorithm. The attack success rate of the proposed adversarial attack method reached 79.9%, which was 16.3% higher than that of the non-channel aware method, verifying the key role of accurate channel estimation in enhancing the effectiveness of the attack.

## 1. Introduction

In the field of communication, channel estimation is a key link to ensure the reliable transmission of signals. However, traditional channel estimation methods have certain limitations when facing complex environments or scenarios with channel sparsity. Although the application of deep learning has brought about some progress, there are still flaws in the network structure and data form. Exploring more accurate and effective channel estimation methods has become an important research direction at present. In today’s technological field, deep learning has been widely applied, yet adversarial attacks have become a major obstacle to its development. The core of adversarial attacks lies in inputting deceptive data into machine learning models, resulting in misclassification of the models. Deep learning adversarial attacks originated in the field of images [1] and then gradually expanded to multiple other fields. During the implementation of adversarial attacks, attackers will perturb the original samples based on their in-depth understanding of the model, such as neural network architecture, learning parameters, loss functions, etc., thereby maximizing the loss function of the classifier and ultimately leading to classification errors. With the advancement of research, adversarial attack algorithms continue to evolve. On the one hand, attackers begin to focus on frequency-domain information because different frequency components have a non-negligible impact on the judgment of the model. By using techniques such as frequency-domain adversarial generative networks [2], spectral attacks [3], and frequency-domain adversarial transport [4], the model can be ingeniously deceived at the frequency-domain level, thereby improving the success rate of attacks. Frequency-domain analysis also helps to deeply understand the model’s response to different frequency components, providing strong guidance for the generation of adversarial samples. This is of great significance for improving the defense mechanism and enhancing the robustness of the model.

In the generation of adversarial samples based on frequency-domain transformation considering channel information, if channel estimation is lacking, the generated adversarial samples are difficult to adapt to the characteristics of the actual communication channel. Due to the failure to consider factors such as multipath propagation, fading, and frequency offset in the channel, in the wireless communication scenario, the adversarial samples may undergo severe distortion after being transmitted through the channel, which greatly reduces the success rate of the attack and makes it impossible to effectively evaluate the security of the model in the real channel environment. Channel estimation can accurately describe the channel characteristics, providing a key basis for the generation of adversarial samples in frequency-domain transformation. This enables the generated samples to optimize the perturbation addition strategy based on the channel conditions, ensuring that they remain deceptive after being transmitted through the channel. Therefore, it is extremely urgent to explore new channel estimation methods, improve the estimation accuracy, and verify their importance in the generation of adversarial samples. This paper proposes an innovative channel estimation method for these problems and verifies it with the aid of adversarial sample generation based on frequency-domain transformation. The specific contributions are as follows:

1. A channel estimation method based on deep learning is proposed. This method fully exploits the potential of deep learning in data processing and feature extraction and is committed to achieving high-precision estimation of sparse channels in OFDM systems.
2. Based on the above channel estimation methods, a channel-aware adversarial sample generation method based on frequency-domain transformation is further proposed to verify the importance of SDRNet channel estimation and significantly improve the effect of adversarial sample generation.
3. The experimental results show that SDRNet significantly outperforms other traditional algorithms in terms of accuracy and robustness in the channel estimation task. Meanwhile, the proposed adversarial attack method also demonstrates a higher attack success rate.

## 2. Background Introduction

Channel estimation is essential for ensuring communication quality, with traditional OFDM-based methods exhibiting distinct advantages and limitations. The least squares (LSs) method [5,6], widely used for its computational simplicity, suffers from severe performance degradation in low-SNR scenarios due to its disregard for noise. Discrete Fourier transform (DFT)-based techniques [7] effectively suppress noise through frequency-domain filtering while maintaining low complexity; however, their performance rapidly deteriorates when frequency offsets exceed 5% of the subcarrier spacing. The linear minimum mean square error (LMMSE) method [8,9], though capable of achieving near-optimal estimation under moderate-to-high SNRs, depends on prior knowledge of channel covariance and exhibits cubic computational complexity, limiting its scalability in large-scale MIMO systems.

Recent developments in deep learning have introduced data-driven alternatives. Deep neural networks (DNNs) [10,11,12] demonstrate strong performance across varying pilot lengths through end-to-end learning, while convolutional neural networks (CNNs) [13] utilize pilot position information to enhance estimation accuracy. Advanced frameworks such as CsiNet and CsiNet-LSTM [14] improve the robustness of CSI feedback, and tensor-train DNNs (TT-DNNs) [15] reduce parameter dimensionality for high-dimensional CSI, albeit with slower convergence. Hybrid approaches that integrate traditional techniques with DNNs [13,16] seek to balance complexity and adaptability, for instance, by leveraging spectral-time averaging to track channel variations.

For sparse channel estimation, traditional algorithms such as orthogonal matching pursuit (OMP) [17] remain prevalent, though their effectiveness heavily relies on accurate sparsity level estimation. Enhanced OMP variants [14,15,18,19] exploit angular sparsity or introduce adaptive mechanisms to improve robustness, often at the cost of increased complexity. In contrast, deep learning-based methods, including CNN-based MIMO-OFDM estimators [20] and DNN-enhanced OTFS systems [21,22] have shown superior performance over OMP, particularly in delay-Doppler domains.

Channel estimation guarantees communication quality and is related to the accuracy and stability of signal transmission. Modulation classification, as the basis of communication systems, is a key prerequisite for subsequent signal processing and information interpretation. The two are closely related and jointly promote the development of communication technology. Under this broad background, the security and stability of communication systems have become key research directions, and adversarial attacks and channel estimation are precisely the core issues among them. In the field of adversarial attacks, many research achievements have been remarkable. In the early days, after Szegedy et al. [23] discovered that deep neural networks were vulnerable to slight adversarial interference, Wu et al. [24,25] proposed the adversarial transformation-enhanced transfer Attack (ATTA), which constructs an adversarial transformation network through adversarial learning to generate adversarial noise and thereby resist the distortion problem caused by the network.

The high-frequency component semantic similarity attack proposed by Luo et al. [26] focuses on the high-frequency noise of the image. Chen et al.’s [27] adversarial attack method based on adversarial generative networks (GANs), adGAN, reduces the performance of intelligent systems by leveraging adversarial generative networks. Xu et al. [28] studied the perturbation pattern analysis of radio frequency signals. Wang et al. [29] utilized Hamiltonian Monte Carlo to generate a series of adversarial samples.

With the in-depth research on the security of communication systems, the correlation between modulation classification and adversarial attacks has gradually emerged. In the context of modulation classification, Zhang et al. [30] evaluated the performance and adversarial sensitivity of Transformer-based neural networks. Manoj et al. [31] introduced multiple training methods to construct robust DNN models and evaluate them. Kotak and Elovicii [32] applied attack assessment to evaluate the vulnerability of the Internet of Things device identification system and discovered new attack methods.

As an important application scenario of communication systems, wireless communication has concrete manifestations and further development in which the above research results are presented. In wireless communication, Sadeghi et al. [33] proposed an adversarial attack method for automatic modulation identification. Lin et al. [34] explored its threats and impacts on automatic modulation recognition. Sandler et al. [35] verified the effectiveness of the attack in the form of external interference. Cohen et al. [36] improved robustness by increasing the noisy training data. Kim et al. [37] studied the channel influence and proposed a channel-aware attack method. Meanwhile, frequency-domain attacks emerged. Guo et al. [38] utilized the low-frequency component attack algorithm, and Sharma et al. [39] discovered different responses of the defense model to high- and low-frequency disturbances. Duan et al. [40] proposed the adversarial attack on DNNs by dropping information (AdvDrop) attack on neural networks.

Despite these advancements, significant challenges remain. Traditional methods struggle in complex and dynamic environments, sparse estimators falter when ideal sparsity assumptions are violated, and deep learning-based approaches still require improved generalization and robustness. To address these issues, we propose a novel channel estimation framework that enhances both accuracy and robustness. Additionally, we introduce a frequency-domain adversarial sample generation method that leverages channel state information to assess the critical role of accurate estimation in communication security. This work bridges the gap between performance and robustness, contributing to the development of reliable and secure wireless communication systems.

## 3. Related Work

### 3.1. Mathematical Model of OFDM Channel Estimation System

**Figure 1:**
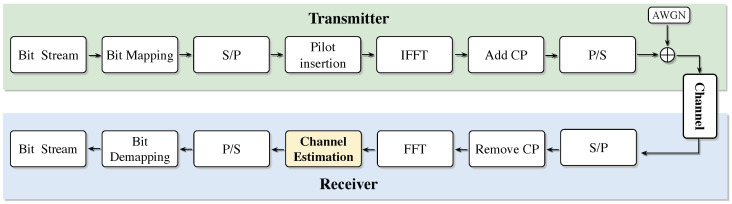
OFDM system model.

The channel estimation system model consists of two parts: the transmitting end and the receiving end. At the transmitting end, the input bit stream is modulated and mapped onto the OFDM subcarriers, and pilot symbols are inserted at specific subcarrier positions for subsequent channel estimation. Then, the frequency-domain signal is converted into a time-domain signal through the inverse fast Fourier transform (IFFT), and a cyclic prefix is inserted. CP is used to resist Inter-symbol interference (ISI), and finally the signal is transmitted through the additive white Gaussian noise (AWGN) channel. At the receiving end, the signal successively removes the cyclic prefix, undergoes fast Fourier transform (FFT) to convert back to the frequency domain, extracts the pilot symbol, and uses the pilot signal for channel estimation, thereby obtaining the channel state information (CSI). Subsequently, the channel influence is eliminated through frequency-domain equalization, the amplitude and phase of the signal are restored, and then the original bit stream is restored through subcarrier demapping and demodulation. The specific process is shown in Figure 1, which shows the training data sources of SDRNet (such as OFDM pilot signals) and the processing flow.

According to the above system model block diagram, we can further establish the above signal processing flow as a mathematical model as follows. Suppose the total number of subcarriers in an OFDM system is *N*, and a subframe contains *I* OFDM symbols in total. For the *i*th OFDM symbol, suppose the *n*th subcarrier symbol on the *i*th symbol is $s_{i}\left(n\right)$, and there is $s_{i}={\left[s_{i}\left(0\right),\ldots ,s_{i}\left(n\right),\ldots ,s_{i}\left(N-1\right)\right]}^{T}$. After OFDM modulation of the frequency-domain symbol through discrete Fourier transform (IFFT):

(1) $$S_{i}=F^{H}s_{i}$$

where $S_{i}\in C^{N\times 1}$ represents the transmitted time-domain sequence and $F^{H}$ is the Fourier transform matrix, specifically expressed as:

(2) $${\left[F\right]}_{n,k}=\frac{1}{\sqrt{N}}\exp\left(-j\frac{2\pi }{N}kn\right)$$

The OFDM transmission model can be further constructed as follows:

(3) $$y_{i}=H_{i}s_{i}+z_{i}$$

where the frequency-domain symbol vector received on the *i*th OFDM symbol block is $y_{i}={\left[y_{i}\left(0\right),\ldots ,y_{i}\left(n\right),\ldots ,y_{i}\left(N-1\right)\right]}^{T}$, $z_{i}$ is the additive white Gaussian noise of the channel, the covariance matrix is $Q_{z}=\sigma^{2}I_{N}$, $\sigma^{2}$ is the noise variance, $I_{N}$ represents the N×N dimensional identity matrix, and $H_{i}\in C^{N\times N}$ represents the frequency-domain response matrix of the channel on:

(4) $$H_{i}=FG_{i}F^{H}$$

where $G_{i}\in C^{N\times N}$ represents the impulse response matrix of the *i*th symbolic time channel, expressed as:

(5) $$G_{i}=\left[\begin{array}{cccccc} h_{i}\left(0,0\right) & 0 & \ldots & h_{i}\left(0,L-1\right) & \ldots & h_{i}\left(0,1\right)\\ h_{i}\left(1,1\right) & h_{i}\left(1,0\right) & 0 & \ldots & \ldots & h_{i}\left(1,2\right)\\ \vdots & \ddots & \ddots & \ddots & \ddots & \vdots \\ 0 & \ldots & 0 & h_{i}\left(N-1,L-1\right) & \ldots & h_{i}\left(N-1,0\right) \end{array}\right]$$

where $h_{i}\left(k,l\right)$ represents the *k*th sampling point of the channel impulse response at the *l*th tap at the *i*th symbol time. Equation (3) can be considered as the mathematical model of baseband signal transmission in the OFDM system, and $H_{i}$ is the parameter to be estimated in the channel estimation link.

For the channel estimation method of OFDM systems, the first step is to estimate the channel response at the pilot symbol position, and then further obtain the channel response on the complete resource block through time-domain interpolation and frequency-domain interpolation. In this paper, it is assumed that the channel impulse response is invariant within an OFDM symbol time, that is, if $h_{i}\left(k,l\right)$ in $G_{i}$ satisfies that the sampling points are equal at different times, then it can be ensured that:

(6) $$h_{i}\left(l\right)=h_{i}\left(0,l\right)=\cdots =h_{i}\left(N-1,l\right)=\frac{1}{N}\sum_{k=0}^{N-1}h_{i}\left(k,l\right)$$

where $h_{i}\left(l\right)$ represents the channel response on the *l*th tap within the time of the *I*th OFDM symbol. Based on this assumption, the channel impulse response matrix $G_{i}$ becomes:

(7) $$G_{i}=\left[\begin{array}{cccccc} h_{i}\left(0\right) & 0 & \ldots & h_{i}\left(L-1\right) & \ldots & h_{i}\left(1\right)\\ h_{i}\left(1\right) & h_{i}\left(0\right) & 0 & \ldots & \ldots & h_{i}\left(2\right)\\ \vdots & \ddots & \ddots & \ddots & \ddots & \vdots \\ 0 & \ldots & 0 & h_{i}\left(L-1\right) & \ldots & h_{i}\left(0\right) \end{array}\right]$$

where $G_{i}$ is obtained by continuously rotating the channel impulse response $h_{i}\left(l\right)$ on different taps. According to the simulation of the properties of the channel frequency-domain response matrix in the previous section and the properties of the discrete Fourier transform, it can be seen that the channel frequency-domain response matrix will become a diagonal matrix as follows:

(8) $$H_{i}=\left[\begin{array}{cccc} H_{i}\left(0\right) & 0 & \ldots & 0\\ 0 & H_{i}\left(0\right) & 0 & \vdots \\ \vdots & 0 & \ddots & 0\\ 0 & \ldots & 0 & H_{i}\left(N-1\right) \end{array}\right]$$

where $H_{i}$ represents the channel response of the channel in the *i*th OFDM symbol time. Further, we simplify Equation (3) based on this assumption, with:

(9) $$y_{i}=X_{i}h_{i}+z_{i}$$

(10) $$X_{i}=diag\left(s_{i}\right)=\left[\begin{array}{cccc} s_{i}\left(0\right) & 0 & \ldots & 0\\ 0 & s_{i}\left(0\right) & 0 & \vdots \\ \vdots & 0 & \ddots & 0\\ 0 & \ldots & 0 & s_{i}\left(N-1\right) \end{array}\right]$$

where $h_{i}=vec\left(H_{i}\right)={\left[H_{i}\left(0\right),H_{i}\left(0\right),\ldots ,H_{i}\left(N-1\right)\right]}^{T}$ is the channel impulse response vector; as shown in the formula, $X_{i}$ represents the channel frequency-domain transmission symbol matrix; function vec(·) represents the operation of extracting the diagonal elements of a matrix and forming a column vector; function diag(·) represents the operation of constructing a diagonal matrix with vector E as the main diagonal element.

### 3.2. Adversarial Disturbance Generation Method

The purpose of adversarial attacks is to add adversarial perturbations to the original benign samples to maximize the loss function value of the classifier and interfere with the classifier’s incorrect judgment. For the input sample *x* and the true label *y*, let the classification model *f* be able to correctly identify sample *x*, then f(x)=y. The goal of the adversarial attack is to generate a subtle adversarial perturbation δ to form an adversarial sample $x^{\prime }=x+\delta$, which can enable the classification model to make a wrong judgment, namely $f(x^{\prime })\neq y$. Attackers usually formulate various strategies for generating perturbations based on the prior knowledge of deep learning models, such as neural network structures, model weights, and model loss functions. For example, FGSM generates adversarial perturbations based on the gradient of the loss function. The adversarial samples are as follows:

(11) $$x^{\prime }=x^{\prime }+\alpha \cdot sign\left(\nabla_{x^{\prime }}J\left(x^{\prime },y\right)\right)$$

where α is the perturbation step size, the adversarial sample $x^{\prime }\in \left[x-\varepsilon ,x+\varepsilon \right]$, *J* represents the loss function, and *y* represents an arbitrary label outside the true sample.

During the attack process, adversarial perturbation ε, also known as perturbation budget, represents the maximum extent of changes that the attacker is allowed to make to benign samples, and δ represents adversarial perturbation. The perturbation budget keeps the perturbations generated by attackers within a displayable and controllable range. Under normal circumstances, after the adversarial sample $x^{\prime }$ is generated, it should exist within the $L_{p}$ norm sphere of the original sample *x*. Commonly used *p* values include 0, 1, 2, and *∞*. In this paper, the focus will be placed on the $L_{2}$ norm, that is, the power limit of the signal. To generate more effective adversarial samples, attackers usually operate with more complex algorithms. For instance, the PGD algorithm gradually adds perturbations to the original data until it successfully misleads the model. The adversarial sample optimization process can be expressed as:

(12) $$x_{t+1}^{\prime }={clip}_{x,\varepsilon }\{x_{t}^{\prime }+\alpha \cdot sign\left(\nabla_{x_{t}^{\prime }}J\left(x_{t}^{\prime },y\right)\right)\}$$

where clip represents the clipping function and J(·) represents the cross-entropy loss.

## 4. Channel Estimation Method Based on Deep Learning

**Figure 2:**
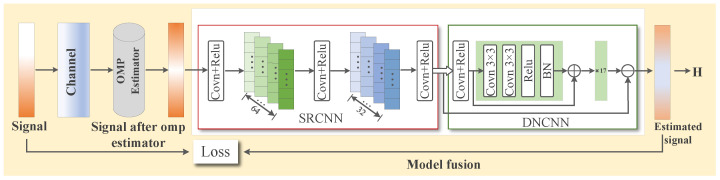
SDRNet channel estimation framework.

**Figure 3:**
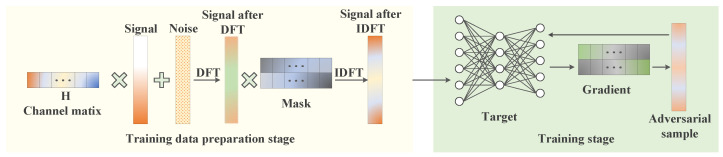
Flowchart of adversarial sample production method based on frequency-domain algorithm and channel awareness.

In this section, firstly, the signal generation, processing and the traditional OMP algorithm are described, and then the network super-resolution denoising network (SRCNN DNCNN network, SDNet) after model fusion is introduced in detail, and the optimized super-resolution denoising residual network (SDRNet) on this basis is introduced. Finally, the generation of the channel-aware adversarial sample algorithm based on frequency-domain transformation was elaborated in detail, verifying the importance and accuracy of SDRNet channel estimation. As shown in Figure 2, it represents the training process of SDRNet, and Figure 3 represents the generation process of adversarial samples.

### 4.1. Residual Channel Estimation Framework for Super-Resolution Denoising Based on Deep Learning

In the SDR channel estimation framework, data preparation should be carried out first and the collected data should be preprocessed. Then, the OMP algorithm is used to estimate the signal, followed by model fusion to obtain the score. Finally, the signal is estimated by integrating the above results to accurately obtain the channel information and achieve efficient and accurate channel estimation.

#### 4.1.1. Date Preparation

In the channel estimation task, in order to simulate the diversified channel environment in a real communication scenario and improve the generalization of the model, we generated signals with different signal-to-noise ratios (SNRs). First, an original signal is generated at the transmitter, which can be represented as a complex sequence:

(13) $$x\left(t\right)=x_{real}\left(t\right)+j\;x_{imag}\left(t\right)$$

where $x_{real}\left(t\right)$ and $x_{imag}\left(t\right)$ represent the real and imaginary components of the signal. Subsequently, we transmit this signal through the channel and receive a signal with added noise at the receiving end. To simulate various SNRs, Gaussian white noise of varying intensity is added to the received signal for each ratio. Specifically, for each SNR, we add noise and generate the corresponding received signal:

(14) $$y_{i}\left(t\right)=x\left(t\right)+z_{i}\left(t\right)$$

where $z_{i}$ represents Gaussian white noise. $y_{i}$ represents the signal received by the receiver.

Next, the generated signals are longitudinally concatenated according to different signal-to-noise ratios to construct a comprehensive training dataset containing multiple channel environments. Suppose *N* signal segments with different signal-to-noise ratios are generated. The operation of vertical splicing can be expressed as:

(15) $$Y=\left[\begin{array}{c} y_{1}\left(t\right)\\ y_{2}\left(t\right)\\ \vdots \\ y_{N}\left(t\right) \end{array}\right]$$

In this way, a matrix containing signal segments with different signal-to-noise ratios was generated, which can simulate the diverse channel conditions in the actual communication environment more comprehensively. In channel estimation, the orthogonal matching pursuit (OMP) algorithm is widely used as an effective sparse signal recovery technique. Therefore, the OMP estimator will be used next to estimate the generated and processed signals. It is applicable to the estimation problem of sparse signals, with low computational complexity and good noise robustness. This algorithm adopts the idea of the greedy algorithm. Essentially, it selects the atoms with the greatest correlation to the signal from a group of atoms called the atomic set. Next, it uses the known atoms to construct estimation coefficients, gradually reduces the residuals, and obtains a sparse signal representation. This algorithm is easy to understand and can accurately restore high-dimensional sparse information.

First, the residual *r* is initialized, and the atom with the highest correlation to the residual is selected from the set of atoms:

(16) $$\lambda_{k}=\underset{i=1,\ldots ,N}{\arg\max}\left|\langle r_{k},A\left(i\right)\rangle \right|,k=0,1,\ldots ,K\;$$

where $\lambda_{k}$ is the *k*th column index determined by the maximum absolute value of the correlation; $r_{0}=b$ represents the residual of the first iteration; A(i) is the *i*th term of *A*, representing the estimated value of the channel matrix.

Next, the column index with the highest correlation is added to the index set $\Lambda^{k}$, and the data corresponding to these indicators in the observation matrix is updated to the reconstructed set of atoms $A_{\Lambda^{k}}$:

(17) $$\Lambda^{k}=\Lambda^{k-1}\cup \left\{\lambda_{k}\right\}$$

(18) $$A_{\Lambda^{k}}=A_{\Lambda^{k-1}}\cup A\left(\Lambda^{k}\right)\;$$

Using the LS method, we obtain an approximate solution:

(19) $$x_{k}=\underset{x}{\arg\min}{∥A_{\Lambda^{k}}x-b∥}_{2}=A_{\Lambda^{k}}^{-1}b$$

Calculate the new approximation of the data and the new residual:

(20) $$r_{k}=b-b_{k}$$

(21) $$b_{k}=Ax_{k}$$

This algorithm selects the vector that best matches the current residual through repeated iterations, and performs orthokerization processing on the selected vectors at each step to gradually construct an approximate solution. When the residuals are small enough or reach the preset sparsity, the iteration terminates. The final solution is a linear combination of all the previously selected vectors. This process employs a greedy strategy to ensure that each step of the choice can minimize the residuals to the greatest extent. It is worth noting that the residuals are always orthogonal to the column space of the selected vectors. When the residuals are not zero and the matrix has a full column rank, the solution of the least square method is unique, thereby ensuring the stability of the algorithm.

#### 4.1.2. Model Integration

In the task of channel estimation, the time-frequency domain characteristics of the channel are complex and changeable, and are vulnerable to noise interference, resulting in limited estimation accuracy. In the field of image processing, super-resolution technology can restore high-resolution details from low-resolution data, and denoising technology can effectively remove noise from images. Since the signal data structure in channel estimation is similar to the image data in features such as multi-channel and two-dimensional distribution, drawing on the SRCNN and DNCNN networks in image processing, the two are integrated and applied to channel estimation. By taking advantage of their abilities to extract details and reduce noise, the accuracy and reliability of channel estimation are improved.

The purpose of SRCNN is to improve the spatial resolution of images. The core idea of SRCNN is to learn the mapping from low-resolution images to high-resolution images through convolutional neural networks. SRCNN consists of three convolutional layers:

(22) $$Y_{srcnn}=f_{3}(w_{3}\times f_{2}(w_{2}\times f_{1}(w_{1}\times X)))$$

where *X* is the input signal, $Y_{srcnn}$ is the output signal, $w_{i}$ represents the convolutional kernel of layer *i*, $f_{i}$ represents the activation function.

SRCNN achieves super-resolution through three layers of convolution. Feature extraction layer $f_{1}$: Map the low-resolution input to the high-dimensional feature space to capture the macroscopic characteristics of the channel response. Nonlinear mapping layer $f_{2}$: Enhance the feature expression ability and restore the high-frequency details lost due to the multipath effect. Reconstruction layer $f_{3}$: Synthesize high-resolution output, whose output dimension matches the input dimension of DNCNN.

The purpose of DNCNN is to remove noise from images, and its core idea is to reduce noise through residual learning. DNCNN consists of multiple convolutional layers and batch normalization layers:

(23) $$Y_{dncnn}=X-F\left(X;\Theta \right)$$

where *X* is the input signal, $Y_{dncnn}$ is the output signal, F(X;Θ) is the convolutional network part of DNCNN, which is capable of extracting the mapping relationship of noise.

In order to integrate these two networks and apply them to channel estimation, this paper designs a model (SDNet) that combines the advantages of both. First, the real and imaginary parts of the complex signal are regarded as two channels of the image, respectively, to construct the input data. Next, the input data is sent to SRCNN to improve the resolution of the signal and obtain the preliminary estimated signal. Finally, the estimated signal output by SRCNN is sent to DNCNN to further remove the noise in the signal and obtain the final accurate channel estimation result. Channel estimation requires restoring the frequency domain details (super-resolution) first and then suppressing the noise (denoising), which is consistent with the image processing flow. The real and imaginary parts of the complex signal are used as dual-channel inputs, retaining the joint phase-amplitude information and enabling the image processing method to be transferred to channel estimation. The key to model fusion lies in the reasonable connection of the two networks, so that the output of the former network can be seamlessly used as the input of the latter network.

### 4.2. The Super-Resolution Denoising Network Model Based on OMP Algorithm

**Figure 4:**
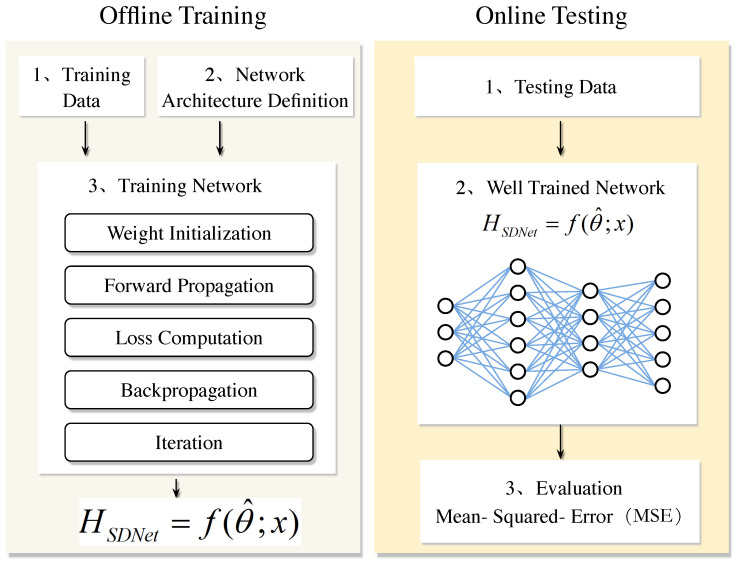
Deep neural network-based channel estimation framework.

**Figure 5:**
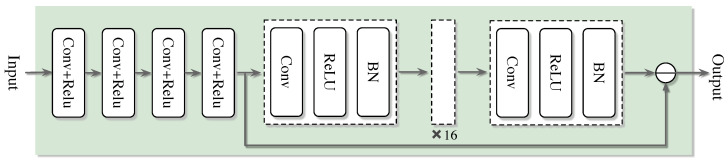
Network architecture of the SDNet.

In the field of signal processing, SRCNN and DNCNN are integrated into the super-resolution denoising network (SDNet) model, aiming to give full play to the super-resolution advantage of SRCNN and the efficient denoising ability of DNCNN, thereby simultaneously improving the accuracy and robustness of channel estimation. Based on this, the SDNet channel estimation framework based on the OMP algorithm was further developed. This framework consists of two stages: discrete training and online estimation. The entire network structure proposed is shown in Figure 4. In the offline training stage, after obtaining the channel estimation result of the OMP algorithm, it is input as the training set into the neural network, and the standard deep neural network training process is used to obtain a well-trained model. Subsequently, in the online estimation stage, the test data is input into the well-trained SDNet to obtain the estimated channel coefficients. Although Figure 5 shows a general process, the proposed algorithm embeds a unique design in each step. In the training network of offline training, we used the specially designed OMP algorithm combined with the deep learning channel estimation algorithm to complete the channel estimation task. During the online testing stage, well-trained networks apply SRCNN and DNCNN to improve the accuracy of channel estimation in our specific scenarios. The overall process is shown in Figure 5 as follows:

#### 4.2.1. Offline Training

The architecture of the SDNet framework consists of the following parts.

Training data generation: prepare a large amount of labeled training data represented as:

(24) $$\left(x,y\right)=\left(x^{\left(1\right)},y^{\left(1\right)}\right),\cdots ,\left(x^{\left(N\right)},y^{\left(N\right)}\right)$$

where *N* represents the number of training samples, each $\left(x^{\left(i\right)},y^{\left(i\right)}\right)$ represents the *i*th (i∈1,2,⋯,N) training samples in the dataset, where $x^{\left(i\right)}$ represents the input data (features) and $y^{\left(i\right)}$ represents the output data used to train the neural network.

According to Equation (3), for the *i*th user, the input data should be the sent signal $S_{i}$ and set the corresponding output as the channel impulse response $H_{i}^{omp}$. Since the inputs and outputs of the neural network must be real numbers, we need to convert the complex-valued matrix into a real-valued matrix.

For the input $S_{i}\in C^{m\times 1}$, we reshape it into a 2D matrix ${S_{i}}^{\prime }\in C^{m_{1}\times m_{2}}$, where $m=m_{1}\times m_{2}$, and both $m_{1}$ and $m_{2}$ are integers. Then, by superimposing the real part and the imaginary part into two channels, the input of OMP-SDNet can be represented as:

(25) x(i)=F{[Re(Si′),Im(Si′)]}∈Rm1×m2×2

where F(·,·) represents conversion to a real function. For the label $X_{reali}$, the real part and the imaginary part are superimposed in turn and reconstructed into a vector with a real value of 2m, which can be expressed as:

(26) y(i)=ReHiomp,ImHiomp∈R2m×1

Network architecture SRCNN→DnCNN→FC: the proposed network is shown in Figure 5. Compared with the classical convolutional layer, the network has better performance in extracting channel features and de-noising. The network architecture adopts a three-level series structure of SRCNN→DnCNN→FC. First, through the three-layer convolution of SRCNN (Conv1: 3 × 3 × 64, ReLU; Conv2: 3 × 3 × 128, ReLU; Conv3: 3 × 3 × 256, ReLU) to achieve super-resolution reconstruction of channel features and extract high-frequency details; subsequently, the five-layer residual module of DnCNN (each module contains 3 × 3 convolution, batch normalization and ReLU activation) is connected to suppress the noise layer by layer and retain the effective signal. Finally, the features are mapped to the channel estimates through the fully connected layer (FC), and the dual-channel results of the real part and the imaginary part are output.

**Figure 6:**
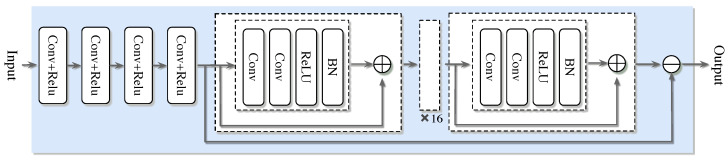
Network architecture of the SDRNet.

First comes the input layer $x^{\left(i\right)}$, which is responsible for receiving and carrying the input data. The second, third, and fourth layers immediately following are convolutional layers, each equipped with a different number of filters with varying sizes of convolution kernels. After each convolutional layer, a rectification linear unit activation function is connected to perform initial feature extraction and transformation of the data. At this point, the obtained intermediate output is saved. Subsequently, the data flows through multiple convolutional layers of size (3 × 3) with the same fill settings. Each Conv layer here is successively connected to a batch normalization layer and a rectification linear unit activation function. Through this series of processing, the noisy image is obtained. Ultimately, subtract the resulting noisy image from the previously saved output, and the difference obtained is the final output result of the entire network. It is worth emphasizing that each connection in the above architecture corresponds to a specific weight. As shown in Figure 6, the output of the entire network can be expressed by the following formula:

(27) $$y_{SDNet}^{\left(i\right)}=f\left(\theta \;;\;x^{\left(i\right)}\right)$$

where θ represents the weight that plays a key role in determining the performance and performance of the model, and f(·) represents the forward propagation function of the whole neural network.

Training network: we will describe in detail how to use the training set to train the proposed OMP-SDNet framework.

First, the weight θ of each layer needs to be randomly initialized. The purpose of this step is to ensure that the network has a different initial state at the beginning of training so that it can learn different feature representations.

Then it enters the forward propagation stage. During this process, each layer of the neural network processes the input data $x^{\left(i\right)}$ in sequence. Firstly, after three convolution and linear activation operations, the purpose is to accurately extract image blocks from the input low-resolution image and map them to the high-resolution space. Then, these high-resolution image blocks are aggregated and integrated to generate the final high-resolution image. The data then passes successively through the module composed of multiple layers of convolution, batch normalization layers, and rectification linear unit activation functions. This module can effectively extract the features contained in the image, and simultaneously complete the tasks of noise removal and image restoration. The output generated by each layer in the above process will be used as the input for the next layer, thus enabling the information to be smoothly transmitted layer by layer until it reaches the output layer. Ultimately, the output result of the network can be represented by $f(\theta \;;\;x^{\left(i\right)})$.

The loss calculation: after each step of forward propagation, we calculate the loss between the predicted output $f(\theta \;;\;x^{\left(i\right)})$ and the actual output $y^{\left(i\right)}$. The goal of training is to minimize losses by adjusting network parameters. In this paper, we use MSE to define the loss function of the network:

(28) $$MSE=\frac{1}{N}\sum_{i=1}^{N}{\left(y_{esti}-y_{real}\right)}^{2}$$

where $y_{esti}$ represents the predicted value, $y_{real}$ represents the actual value, and *N* represents the sample size. Our training goal is to find a well-trained set to $\hat{\theta }$ to minimize the loss function, so the optimization problem in this paper can be expressed as:

(29) $$\min_{\theta }L_{SDNet}\left(\theta \right)=\frac{1}{N}\sum_{i=1}^{N}{∥f\left(\theta \;;\;x^{\left(i\right)}\right)-y^{\left(i\right)}∥}^{2}$$

In neural network training, we use backpropagation to calculate the loss gradients and apply the Adam optimization algorithm to update the network parameters according to these gradients. When training, the data are divided into small batches and processed in multiple cycles. Each cycle, the model predicts by forward propagation, calculates MSE losses, backpropagation acquires gradients, and updates parameters to minimize losses. After the training is completed, a model with optimal parameters can be obtained, which can accurately predict new data. The final trained model can be expressed as:

(30) $$y_{SDNet}=f\left(\hat{\theta }\;;\;x\right)$$

where *x* represents the input data and $y_{SDNet}$ represents the output data, which needs to be converted into complex value channel information $H_{SDNet}$. We can express the estimated channel matrix $H_{SDNet}$ as:

(31) $$H_{SDNet}=\frac{y_{SDNet}}{X_{esti}}$$

#### 4.2.2. Online Estimation

In the online estimation stage of channel estimation, the optimal parameter model obtained after offline training of the neural network will be deployed to the wireless communication system. In the process of online estimation, the system will first capture the real-time received signal and convert it into a real-valued matrix suitable for neural network processing according to the formula. These real-valued matrices are then fed into a trained neural network, SDNet, and the predicted channel information is obtained through forward propagation.

However, with the increase in network depth in the OMP-SDNet, the problem of over-fitting or gradient disappearance may be encountered, resulting in a poor estimation effect. Therefore, in order to further improve the accuracy of channel estimation, we will propose a neural network architecture based on ResNet in the next section.

### 4.3. The Super-Resolution Denoising Residual Network Model Based on OMP Algorithm

In order to further improve the accuracy of channel estimation, a channel estimation framework was developed. This framework integrates the residual network (Resnet) module based on SDNet and is denoted as SDRNet.

Here, the ResNet module is integrated into the channel estimation framework. Adding residual connections in the part after three convolutions can help the network better learn the residuals to solve the problem of vanishing gradients and thereby improve the denoising performance.

The improved channel estimation framework OMP-SDRNet also includes two stages: (1) offline training and (2) online estimation. The entire workflow is shown in Figure 6. Similar to the SDNet framework, the training data generation method of SDRNet is the same, but their network architectures are different. The ResBlock component of the super-resolution denoising residual network (SDRNet) is a key architectural innovation. This section focuses on the design, functions, and impacts of ResBlocks, which are integrated to solve the vanishing gradient problem in deep networks and enhance the channel estimation performance in OFDM systems. The ResBlock in Figure 6 consists of a skip connection and two convolutional sub-layers, aiming to facilitate deep learning by enabling the network to learn residual mappings rather than direct input–output relationships. The detailed structure is as follows. Convolutional layer: Two consecutive 3 × 3 convolutional layers, filled to maintain the dimension of the feature map. After each convolutional layer, there is batch normalization (BN) to standardize activation and accelerate training, and there is also a ReLU activation function to introduce nonlinearity. Skip the connection: Directly connect the input of the block to the output of the convolutional layer, allowing the network to learn the residual between the input and the expected output. This design ensures that the network can learn incremental improvements (residuals) instead of reconstructing the entire signal from scratch, reducing gradient vanishing. In Figure 6, after the initial feature extraction layer based on srcnn, multiple Resblocks are cascaded in the denoising module of SDRNet. The workflow is as follows. Feature extraction: The SRCNN module (the first few layers) processes the OFDM pilot signal to restore the high-resolution channel features. Residual denoising: Cascades ResBlocks receive features from SRCNN and gradually suppress noise by learning residuals (i.e., the difference between noisy features and clean channel responses). Channel reconstruction: The output of ResBlocks is fed into the final convolutional layer to generate an estimated channel matrix, leveraging denoising and enhanced features. The skip connections in ResBlocks bypass the deep convolutional layers, ensuring that the gradients propagate effectively through the network. Compared with the non-residual SDNet that lacks such connections, this allows SDRNet to train deeper architectures. Noise suppression: By focusing on residual learning, SDRNet more effectively separates noise from the effective channel features. For instance, in low signal-to-noise ratio scenarios, ResBlocks can distinguish between multipath fading (expected signal) and Gaussian noise (residual), thereby generating clearer estimates. At this time, the output result can be expressed as:

(32) $$x^{\left(j\right)}=F_{conv}[x^{\left(i\right)}\left(w^{\left(i\right)}\right)]$$

The improved channel estimation framework OMP-SDRNet also includes two phases: (1) offline training and (2) online estimation. The whole workflow is shown in Figure 4. where $F_{conv}\left(\cdot \right)$ represents the convolution operation; $x^{\left(i\right)}$ indicates the input training data; $w^{\left(i\right)}$ represents the set of parameters of the three convolutions operation processes.

Next, the results after three convolutions and linear activation are used as the input of the first Residual block. The basic structure of the residual block consists of two parts: direct mapping and residual mapping, and the output of each block can be expressed as:

(33) $$y^{\left(j\right)}=F\left[x^{\left(j\right)}\left(w^{\left(j\right)}\right)\right]+x^{\left(j\right)}$$

where *j* represents the number of residual blocks, $x^{\left(j\right)}$ and $y^{\left(j\right)}$ are the input and output of the residual blocks, and $w^{\left(j\right)}$ is the set of parameters for these operations.

Residual mapping $F\left[x^{\left(i\right)}\left(w^{\left(i\right)}\right)\right]$ usually consists of two or three convolution layers, each of which is followed by batch normalization (BN) and ReLU activation functions.

The final output result can be expressed as:

(34) $$y_{SDRNet}=y^{\left(j\right)}$$

where *j* represents the value of the last residual block, which needs to be converted into complex-valued channel information $H_{SDRNet}$. We can express the estimated channel matrix $H_{SDRNet}$ as:

(35) $$H_{SDRNet}=\frac{y_{SDRNet}}{X_{esti}}$$

Through adequate training, SDRNet successfully introduced the concept of residual learning into the task of channel estimation. The structure of the residual block allows the network to learn the residual representation between the input and output, thereby simplifying the learning difficulty and avoiding the problem of vanishing gradients. The integration method adopted in this paper can significantly improve the denoising performance of neural networks. The loss calculation and backpropagation sections are similar to the SDNet framework proposed in the previous section and will not be elaborated on in detail here.

## 5. Channel Aware Adversarial Sample Generation Method Based on Frequency Domain Transformation

Channel estimation guarantees communication quality and is related to the accuracy and stability of signal transmission. Modulation classification, as the basis of communication systems, is a key prerequisite for subsequent signal processing and information interpretation. The two are closely related and jointly promote the development of communication technology. Under this broad background, the security and stability of communication systems have become key research directions, and adversarial attacks and channel estimation are precisely the core issues among them.

In recent years, the research on adversarial attacks based on frequency-domain information has revealed the frequency-domain sensitivity characteristics of deep learning from two dimensions: attack methods and model mechanisms. At the level of attack methods, researchers have found that low-frequency components and high-frequency components have the same influence on model decision-making. Although the existing defense mechanisms can effectively suppress high-frequency disturbances, there are still significant loopholes in the defense against low-frequency disturbances.

At the model mechanism level, research shows that deep neural networks have significant frequency-domain perception preferences. On the one hand, the model can capture high-frequency features that are difficult for humans to detect, but is extremely sensitive to high-frequency noise. On the other hand, network decision-making overly relies on spectral amplitude information while ignoring the robustness characterization of phase characteristics. Further research has found that the key discrimination frequency bands of samples of different categories are specific. This category difference in frequency sensitivity provides an opportunity for targeted frequency-domain attacks. The current defense methods enhance robustness through strategies such as high-frequency noise suppression and phase protection. However, breakthroughs are still needed in cross-band attack defense and dynamic spectrum registration, which points out the direction for subsequent research.

From the above content, it can be known that different frequency components in the existing research contribute differently to the model decision-making. Then, different frequency components also have guiding significance for the generation of adversarial samples. Based on this, this paper proposes an adversarial sample generation method based on channel awareness for frequency-domain transformation. At the same time, it can verify the importance of accurate channel estimation in adversarial attacks on communication systems. Provide new solutions for the security and reliability of communication systems.

In this section, the Fourier transform is adopted to transform the signal from a continuous time series signal in the time-domain to the frequency domain for analysis. The specific mathematical expression is as follows:

(36) $$F\left(w\right)=\int_{-\infty }^{\infty }f(t)\times e^{-jwt}dt$$

(37) $$e^{-jwt}=\cos\left(wt\right)-j\times \sin\left(wt\right)$$

where $e^{-jwt}$ represents an odd function. When both the function and its Fourier transform undergo discretization processing, the discrete Fourier transform (DFT) can be obtained. For radio signals that are usually modulated by I/Q modulators, the I/Q two-channel signals can be expressed as:

(38) $$x\left(t\right)=I\left(t\right)+jQ\left(t\right)=e^{j\varphi (t)}$$

For a signal with a sampling length of *L*, it can be expressed as:

(39) $$x=\left[\begin{array}{c} I\\ Q \end{array}\right],\;\left\{\begin{array}{c} I=\left[i_{1},i_{2},\ldots ,i_{L}\right]\\ Q=\left[q_{1},q_{2},\ldots ,q_{L}\right] \end{array}\right\}$$

Therefore, the discrete Fourier transform of the modulated signal of length *L* in the I/Q two channels can be expressed as:

(40) $$X\left(m\right)=\sum_{n=0}^{L-1}x\left(n\right)\cdot e^{j\frac{2\pi }{L}mn}$$

Correspondingly, the frequency-domain signal can be converted into a time-domain signal through the inverse discrete Fourier transform, and its expression is:

(41) $$x\left(n\right)=\frac{1}{N}\sum_{m=0}^{L-1}X\left(m\right)\cdot e^{j\frac{2\pi }{L}mn}$$

Based on the existing research and analysis, if the sensitivity of different frequency components of the sample to model recognition can be explored and malicious disturbances can be generated guided by this, the generated adversarial samples will be more targeted and threatening. For this purpose, this section proposes a frequency-domain signal processing method based on random masks. By inputting samples under different frequency distributions and observing the feedback of the model, the specific operation process T(x) can be expressed as:

(42) $$\begin{array}{c} T(x)=IDFT((DFT(x)+DFT(\xi ))⊙M)\\ \;\;\;=IDFT(DFT(x+\xi )⊙M) \end{array}$$

where DFT(·) and IDFT(·) represent the discrete Fourier transform and inverse discrete Fourier transform, $\xi ∼N(0,\delta^{2})$ represents random noise obeying a Gaussian distribution. M∼U(1−ρ,1+ρ) represents the mask matrix, whose elements are random samples in a uniform distribution, and ⊙ represents the Hadamar product, which is the product of each element in matrix operations. The frequency domain conversion process F can be seen as shown on Figure 2.

Furthermore, the signal data processed by Equation (42) are input into the target classification model and the model gradient information *g* is obtained. In order to obtain more reliable frequency-domain sensitivity information, in this section, the process *T* is selected for *N* times, that is, the noise $\xi_{i}$ and mask $M_{n}$ generated in each round, and a set of gradient information $M_{n}$ is obtained, where n=1,2,…N. After completion, continue to sum the *N* gradients and calculate the average value. The total gradient information highlights the sensitive regions of the model for robust rows and key features, thereby guiding adversarial samples to be generated in a more threatening direction. Each generated mask element follows a uniform distribution. In this section, the weights of the time-domain and frequency-domain components of each gradient are set to 1.

Finally, the obtained gradient information is combined with the attack algorithm in Equation (12) to generate adversarial samples. To sum up, the complete adversarial attack algorithm based on frequency-domain transformation can be summarized as the following formula:

(43) $$\begin{array}{c} x_{i+1}^{\prime }=\\ clip_{x,\varepsilon }\left\{x_{i}^{\prime }+\alpha \cdot sign\left(\frac{1}{N}\sum_{n=1}^{N}\nabla_{x_{i}^{\prime }}J\left(T\left(x_{i}^{\prime }\right),y,H\right)\right)\right\} \end{array}$$

where ε represents the limit of the disturbance $L_{\infty }$, α=ε/I, *I* represents the number of iterations $sign\left(\cdot \right)$ represents the sign function, α represents the iteration step size, $J\left(\cdot \right)$ represents the target model loss function, and *H* represents the channel matrix information.

Next, consider a wireless communication system composed of one transmitter, *m* receivers, and one adversary. All nodes are equipped with an antenna and operate on the same channel. Each receiver uses a neural network to classify the signals it receives into the modulation type used by the transmitter. Meanwhile, the opponent transmits disturbance signals through the air, deceiving the classifier on the receiver into making mistakes in modulation classification, thereby enabling the attack to succeed.

The deep neural network classifier at the *i*th receiving end is denoted as $f^{\left(i\right)}\left(\cdot \;;\theta_{i}\right):\chi \to R^{C}$, where a represents the parameters of the neural network at the *i*th receiving end and *C* is the number of modulation types. Here, $\chi \subset C^{p}$, and *p* is the dimension of the complex input (in-phase/orthogonal component), which can also be expressed as the concatenation of two real input numbers. The classifier $f^{\left(i\right)}$ assigns the modulation type ${\hat{l}}^{\left(i\right)}\left(x,\theta_{i}\right)={\mathrm{argmax}}_{k}f_{k}^{\left(i\right)}\left(x,\theta_{i}\right)$ to each input x∈χ, where $f_{k}^{\left(i\right)}\left(x,\theta_{i}\right)$ is the output of the *i*th classifier for the *k*th modulation type.

The channel from the transmitting end to the *i*th receiving end is denoted as $h_{tr_{i}}$, and the channel from the opponent to the receiving end is denoted as $h_{ar_{i}}$. The vector forms are represented by $h_{tr_{i}}={\left[h_{tr_{i},1},h_{tr_{i},2},\ldots ,h_{tr_{i},p}\right]}^{T}\in C^{p\times 1}$ and $h_{ar_{i}}={\left[h_{ar_{i},1},h_{ar_{i},2},\ldots ,h_{ar_{i},p}\right]}^{T}\in C^{p\times 1}$. When there is no adversarial attack, the transmitting end sends signal *x*, and the signal received by the *i*th receiving end is $r_{tr_{i}}=H_{tr_{i}}x+n_{i}$. When there is an adversarial attack, if the attacker transmits a perturbation signal δ, the signal at the *i*th receiving end is $r_{ar_{i}}\left(\delta \right)=H_{tr_{i}}x+H_{ar_{i}}\delta +n_{i}$, where $H_{tr_{i}}$ and $H_{ar_{i}}$ are diagonal matrices as mentioned earlier, and $n_{i}$ is Gaussian noise.

Suppose the adversarial disturbance δ and the transmitting signal *x* are synchronously superimposed at the receiving end to ensure the effectiveness of the attack. To achieve the concealment and energy efficiency of the attack, the adversarial perturbation δ needs to satisfy the power constraint ${∥\delta ∥}_{2}^{2}\leq P_{\max}$, where $P_{\max}$ is the preset maximum perturbation power budget. The attacker needs to design a universal perturbation δ for the input signal *x* and all receiver classifiers $f^{\left(i\right)}$ by solving the following optimization problems (44):

(44) $$\begin{array}{c} \underset{\delta }{\arg\min}\;{∥\delta ∥}_{2}\\ \mathrm{subject}\mathrm{to}\;{\hat{l}}^{\left(i\right)}\left(r_{tr_{i}},\theta_{i}\right)\neq {\hat{l}}^{\left(i\right)}\left(r_{ar_{i}}\left(\delta \right),\theta_{i}\right)\;i=1,2,\ldots ,m\\ \;\;\;\;{∥\delta ∥}_{2}^{2}\leq P_{\max} \end{array}$$

In optimization problems (44), the objective is to minimize the perturbation power (minimize the $L_{2}$ norm) to ensure that the perturbation power does not exceed the budget $P_{\max}$ while satisfying all receiver classification errors. It should be noted that due to the complexity of the decision boundary of deep neural networks, the optimal solution may not be obtained at point ${∥\delta ∥}_{2}^{2}=P_{\max}$.

The analysis will be carried out from the single-receiver scenario (m = 1), and the receiver index *i* will be omitted to simplify the expression. For targeted attacks, the attacker designs the perturbation δ by minimizing the loss function $J\left(r_{ar},y,\phi \right)$. Based on the fast gradient method (FGM), the loss function can be linearly approximated as $J\left(r_{ar},y,\phi \right)\approx J\left(r_{tr},y,\phi \right)+{\left(H_{ar}\delta \right)}^{T}\nabla_{x}J\left(r_{tr},y,\phi \right)$. Minimization is achieved by setting $H_{ar}\delta =-\beta J\left(r_{tr},y,\phi \right)$, and β is the scaling factor used to constrain the adversarial disturbance power to $P_{\max}$.

In the MRPP attack [37], the attacker maximizes the perturbation power at the receiving end by selecting perturbations and analyzes the impact of this power on the classifier decision-making process. To achieve this goal, attackers need to make full use of the channel characteristics between the attacker and the receiving end. Specifically, if the target attack disturbance $\delta^{target}$ is multiplied by the conjugate ${h_{ar}}^{*}$ of channel $h_{ar}$, the received power can be maximized along the channel direction. After being transmitted through the channel, the disturbance power at the receiving end becomes ${∥h_{ar}∥}_{2}^{2}\delta^{target}$. Through this operation, the adversarial attack not only maintains the consistency of the perturbation direction with the channel but also maximizes the transmission efficiency of the perturbation energy through the channel gain. Ultimately, the attacker needs to generate targeted perturbations for all possible modulation types and calculate the scaling factor to meet the power constraints of the opponent. The calculation of the scaling factor β has been obtained from reference [33] and will not be elaborated here.

Based on the above derivation and combined with the methods mentioned in the previous section, the adversarial sensing adversarial perturbation generation algorithm based on frequency-domain transformation can be obtained. The specific details are given in Algorithm 1.
**Algorithm 1** Adversarial example generation algorithm channel sensing and frequency-domain transformation (FTHA).**Input** **:**Classification model *f* with parameters ϕ, clean sample *x*, true label *y*, $L_{\infty }$ norm bound ϵ, iteration count *I*, frequency transformation count *N*, coordination factor ρ, noise ξ with standard deviation σ, mask *M*, $H_{ar}$ representing channel matrix to receiver, $H_{ar}^{*}$ representing conjugate of channel matrix**Output** **:**Adversarial sample $x^{\prime }$ 1:α=ϵ/I, $x_{i}=x$, ξ follows Gaussian distribution $\left(0,\delta^{2}\right)$, *M* follows uniform distribution    (1−ρ,1+ρ) 2:**for** i=1 to *I* **do** 3:    **for** n=1 to *N* **do** 4:         Random frequency transformation:              $T\left(x^{\prime }\right)=IDFT\left(DFT\left(x+\xi \right)⊙M\right)$ 5:         Gradient calculation: $g_{n}=\nabla_{{x_{i}}^{\prime }}J\left(T\left({x_{i}}^{\prime }\right),y;\phi \right)$ 6:    **end for** 7:    Compute average gradient: $g_{n}=\frac{1}{N}\sum_{n=1}^{N}g_{n}$ 8:    Channel information perturbation:        $\delta^{t\arg et}=\frac{H_{ar}^{*}\nabla_{{x_{i}}^{\prime }}J\left(T\left({x_{i}}^{\prime }\right),y;\phi \right)}{{∥H_{ar}^{*}\nabla_{{x_{i}}^{\prime }}J\left(T\left({x_{i}}^{\prime }\right),y;\phi \right)∥}_{2}}$ 9:    ${x_{i+1}}^{\prime }=clip\left(clip_{x,\varepsilon }\left({x_{i}}^{\prime }+\alpha \cdot H_{ar}\cdot \delta^{t\arg et},-1\right),1\right)$10:**end for**11:$x^{\prime }=x_{i}^{\prime }$12:**return** $x_{i}^{\prime }$

## 6. Performance Indicators

### 6.1. Channel Estimation Performance Indicators

In order to evaluate the performance of the proposed OMP-SDRNet frameworks in OFDM systems, we choose the mean square error and the bit error rate to measure. Firstly, the MSE performance of channel estimation is analyzed as follows:

(45) $$MSE=\frac{1}{N}\sum_{i=1}^{N}{\left(H_{esti}-H_{real}\right)}^{2}$$

where $H_{esti}$ represents the estimated channel information, i.e., $H_{SDNet}$ and $H_{SDRNet}$, and $H_{real}$ represents the actual channel information. The BER performance of channel estimation is analyzed as follows:

(46) $$BER=\frac{Bit_{err}}{Bit_{sum}}$$

where $Bit_{err}$ represents the number of incorrect bits and $Bit_{sum}$ represents the total number of bits.

### 6.2. Adversarial Attack Performance Indicators

To compare the adversarial attack methods in this section, the adversarial performance is measured by the misclassification rate, the average confidence of the wrong class prediction, the average confidence of the correct class prediction, the perturbation-to-signal ratio and the $L_{2}$ norm.

The misclassification ratio (MR) is the most important attribute in adversarial attacks. In untargeted attacks, MR is defined as the proportion of all samples that are successfully misclassified into any class, and therefore can also be called the attack success rate. Specifically as follows:

(47) $$MR=\frac{1}{N}\sum_{i=1}^{N}count(f(x_{i}^{a})\neq y_{i})$$

where $f\left(\cdot \right)$ represents the classification model, there are f(x)=y, *x* represents the normal sample, and $x^{a}$ represents the adversarial sample. In the experiment, all the adversarial samples were from the samples that were correctly classified by the target model.

The average confidence of adversarial class (ACAC) represents the model’s confidence in predicting the class of the sample after perturbation as follows:

(48) $$ACAC=\frac{1}{n}\sum_{i=1}^{n}Q{\left(x_{i}^{a}\right)}_{f(x_{i}^{a})}$$

where *Q* is the softmax layer output of the classifier *f*, there exists $f\left(x\right)=\arg\max_{j}Q{\left(x\right)}_{j}$, and $Q{\left(x\right)}_{j}$ represents the probability of the *j*th class.

The average confidence of true class (ACTC) represents the model’s confidence in the correct class of the sample after the attack. ACAC is used to evaluate the degree to which the adversarial sample deviates from the true class as follows:

(49) $$ACTC=\frac{1}{n}\sum_{i=1}^{n}Q{\left(x_{i}^{a}\right)}_{y_{i}}$$

The $L_{2}$ norm is the most frequently used norm for calculating Euclidean distances and is also often employed as the regularization term for optimizing the objective function.

The perturbation-to-noise ratio is the ratio of perturbation power to noise power, which can be calculated through perturbation-to-signal ratio (PSR) and signal-to-noise ratio (SNR), as follows:

(50) $$PNR=\frac{p_{per}}{p_{n}}=PSR\times SNR$$

where $p_{per}$ represents the disturbance power and $p_{n}$ represents the noise power.

## 7. Simulation Result

### 7.1. Channel Estimation

In this section, the experiments for verifying the effectiveness of the proposed method are described in detail, and the experimental results are presented. This experiment is built based on the OFDM communication system. The application scenario is set as transmission by a single antenna and reception by a single user. The channel model selected is the Rayleigh channel. The Pytorch toolkit is selected as the deep learning development tool, and all deep learning models are trained on NVIDIA Tesla V100-PCIE. We make full use of its powerful functions of deep learning model construction and training to provide strong support for the implementation of the SDNet and SDRNet frameworks. The sample size is set at 2000, and the signal data are generated by using the QPSK modulation method. To obtain the test set, the output of the OMP algorithm is used as the input data of the neural network, thereby providing basic data support for the subsequent network performance evaluation.

In the network compilation stage, the Adam optimizer is selected for training because it performs well in parameter optimization and can effectively adjust network parameters to improve model performance. The learning rate is set to 0.0005 to maintain a stable update during the training process. The mean square error is adopted as the loss function throughout the training process, and the loss of the validation set is taken as the key evaluation index to measure the training effect and generalization ability of the model. Furthermore, in all experiments, other parameters were kept uniform, and the number of loop iterations was fixed at 100 times to ensure the scientificity and comparability of the experimental results, facilitating the accurate evaluation of the performance of different methods. Secondly, the default system parameters used in the channel simulation are summarized as shown in Table 1.

**Figure 7:**
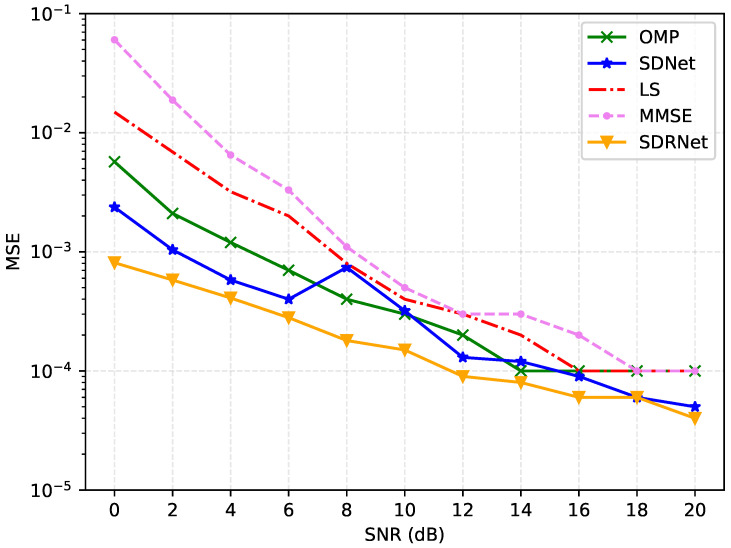
Comparison of MSE results for different channel estimation methods.

**Figure 8:**
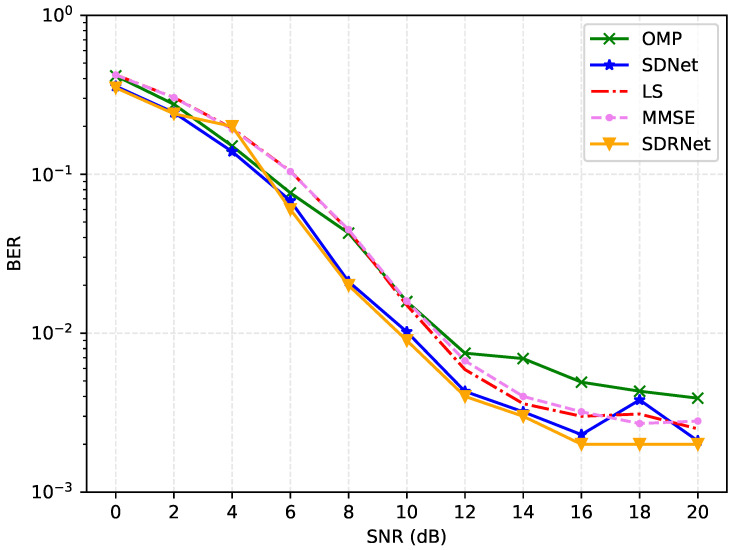
Comparison of BER results for different channel estimation methods.

Figure 7 and Figure 8 present a detailed analysis of the mean square error (MSE) and bit error rate (BER) of different channel estimation algorithms under a signal-to-noise ratio of 0–20 dB. The results show that the MSE and BER of all methods decrease as the signal-to-noise ratio increases. This is because a higher transmission power can effectively resist noise interference.

Based on the LS algorithm in low SNR and poor performance, a high signal-to-noise ratio is better. This is because the LS algorithm regards the channel as a definite but unknown constant and uses linear estimation, which is extremely sensitive to noise. In comparison, the performance of the MMSE algorithm is slightly better because it takes into account the prior statistical characteristics of the channel and uses weighted coefficients for linear estimation. The OMP algorithm estimates by gradually selecting the most relevant sparse channel components, which can effectively utilize the channel sparsity and have a higher estimation accuracy than the previous two.

Different from traditional algorithms, the framework proposed in this paper adopts a nonlinear and multi-layer neural network structure, which can estimate more complex channels. Overall, SDNet and SDRNet perform exceptionally well across the full signal-to-noise ratio range, with their MSE significantly lower than that of OMP, LS, and MMSE. Among them, SDRNet performed the most prominently. At 20 dB, the MSE was as low as 0.00004, showing significant advantages. This is attributed to SDRNet integrating multiple residual blocks on the basis of SDNet, retaining the output results of the previous module, and making full use of the features. Therefore, regardless of the environment of low signal-to-noise ratio (0–4 dB), medium signal-to-noise ratio (6–12 dB), or high signal-to-noise ratio (14–20 dB), the MSE and BER of SDRNet remain at a relatively low level, highlighting its superiority and robustness in channel estimation. These data strongly prove the effectiveness of SDNet and SDRNet in channel estimation, and further verify the performance advantages of the proposed method.

**Figure 9:**
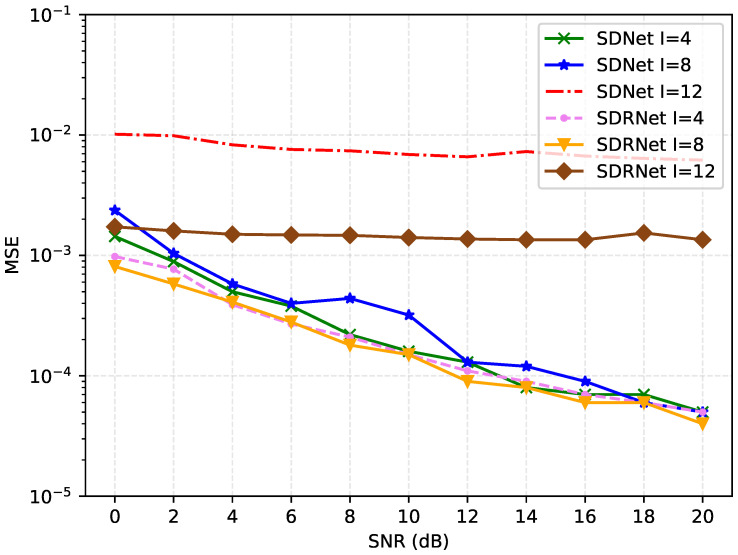
Comparison of MSE results for different pilot spacing I.

Figure 9 shows the MSE comparison of the proposed method under the same number of pilots, the same channel sparsity, and different pilot interval schemes. For SDNet, as the pilot interval I increases, the MSE initially shows an upward trend (from I = 4 to I = 8), and then significantly increases at I = 12. This indicates that under a smaller pilot interval, SDNet can better utilize the pilot signal for channel estimation. However, when the pilot interval is too large, the performance will decline significantly.

For SDRNet, MSE decreases slowly with the increase in the pilot interval I, but the overall change is not significant. This indicates that SDRNet is relatively insensitive to the change in pilot intervals and can maintain relatively stable performance under different pilot intervals. Under most pilot intervals, the MSE of SDRNet is generally lower than that of SDNet, especially at larger pilot intervals (such as I = 12), the performance advantage of SDRNet is more obvious. This indicates that SDRNet may have adopted more effective algorithms or structures in channel estimation and can provide better performance under different pilot intervals. Moreover, the MSE curve of SDRNet is smoother, showing better stability and robustness.

**Figure 10:**
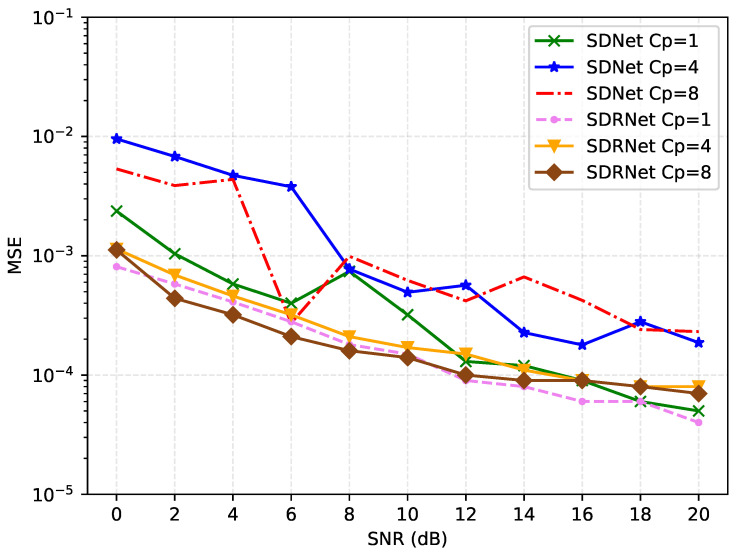
Comparison of MSE results for different cyclic prefix lengths (CP).

Figure 10 shows the comparison of MSE performance of different pilot quantity schemes under the same pilot interval and channel sparsity. For SDNet, as the number of pilots increases (Nc = 8 to 32), the MSE decreases significantly, mainly due to the cyclic prefix (CP) improving signal continuity and reducing time-domain aliasing. However, when the pilot is too much (Nc = 64), the MSE slightly rebounds, which might be due to an increase in pilot overhead or interference. The MSE of SDRNet continuously and smoothly decreases with the increase in pilot frequency, indicating that it can utilize pilot resources more efficiently. Overall, the variation range of MSE in the two methods is limited, indicating that they are not sensitive to the number of pilots, which is conducive to reducing the system overhead.

**Figure 11:**
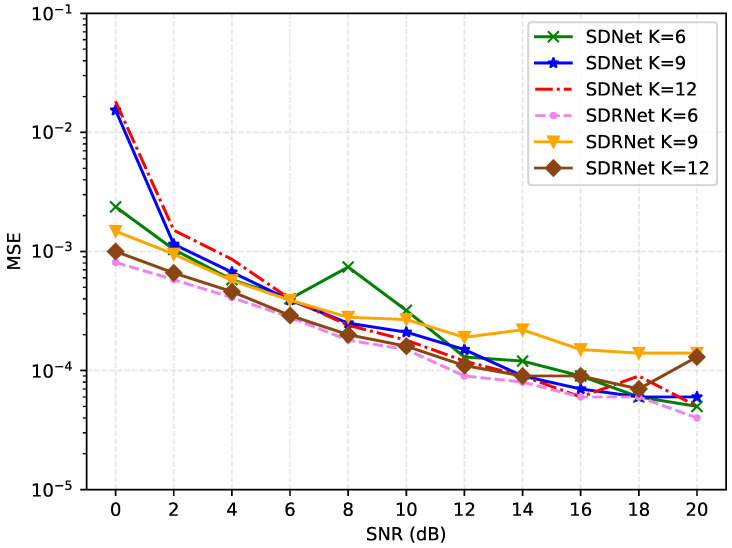
Comparison of MSE results for different channel sparsity levels (K).

Figure 11 compares the MSE performance changes in SDNet and SDRNet under different channel sparsity (K = 3, 6, 9). When the sparsity of SDNet increases from K = 3 to K = 6, the MSE slightly decreases, indicating that a moderate increase in sparsity is conducive to improving the accuracy of channel estimation. However, when the sparsity further increases to K = 9 and K = 12, the MSE rises significantly, indicating that an excessively high sparsity will make the channel overly complex and reduce the estimation performance. In contrast, the MSE of SDRNet slightly decreased from K = 3 to K = 6, and then remained stable, demonstrating strong robustness to changes in sparsity. This indicates that SDRNet can better adapt to channel environments with different sparsity, while the performance of SDNet depends more on the reasonable selection of sparsity.

### 7.2. Adversarial Sample Generation Based on Frequency Conversion and Channel Awareness

To verify the effectiveness of the FTHA method, this study conducted a phased and progressive experiment for verification. Firstly, in the benchmark environment of the ideal channel, by comparing with traditional attack methods, the advantages of FTA in terms of perturbation efficiency and concealment are verified. Further, we introduce multi-dimensional channel conditions to construct an adversarial attack and defense test platform in real communication scenarios, and compare and analyze the performance differences between FTHA and the classic channel-aware attack strategies, as well as traditional attack methods considering channel conditions in key indicators such as attack success rates.

This section mainly introduces the specific implementation process and testing of adversarial samples based on frequency-domain transformation with channel awareness. As a result, the task experiments on signal modulation recognition in this chapter were conducted in the RadiOML 2016.10A public dataset Proceed. RadioML2016 is an open-source benchmark dataset in the field of wireless communication, mainly used for modulation identification tasks. This dataset was released by Tim O’Shea et al. in 2016, aiming to provide a standardized test platform for the performance evaluation of deep learning models in complex wireless signal processing tasks. Its core objective is to promote the application of machine learning in fields such as wireless communication security and spectrum sensing by simulating the signal characteristics in real communication environments. It includes 11 modulation methods, covering common digital modulations (such as BPSK, QPSK, 16QAM, 64QAM) and analog modulations (such as AM and FM). Each sample is IQ data In complex form (in-phase and quadrature components), with a sampling length of 128 time points, which can completely capture the time-domain characteristics of the signal. By adding Gaussian white noise (AWGN) with different signal-to-noise ratios, the noise interference in actual communication is simulated.

Furthermore, some data introduce channel distortions such as multipath fading and frequency shift. The dataset contains approximately 2.2 million samples in total, which are evenly distributed hierarchically by modulation type and SNR to ensure the comprehensiveness of training and evaluation. It is usually divided into the training set (80%), the validation set (10%), and the test set (10%), supporting the model development of supervised learning tasks.

For the target task, namely the model of automatic modulation recognition, the commonly used models of automatic modulation recognition selected in the experiments of this chapter are as follows:

(1) CNN1D: Modulation recognition model based on one-dimensional convolutional residual network;

(2) CNN2D: Modulation recognition model based on two-dimensional convolutional neural networks.

Firstly, without considering the channel information, in order to verify the effectiveness of the proposed adversarial attack algorithm based on frequency-domain transformation, this section selects multiple adversarial attack algorithms as baselines and compares them with the proposed method:

(1) FGSM: It generates adversarial samples by using the gradient information of the input data, and implements attacks by adding or subtracting the sign of the gradient on each element of the input data and multiplying it by a tiny perturbation.

(2) PGD: During the iterative process, adversarial samples are constructed by maximizing the loss function within the perturbation range to ensure that the generated samples have stronger interference.

(3) BIM: By applying minor disturbances to the original input and conducting multiple iterations to generate adversarial samples, the loss function is maximized within the disturbance range in each iteration.

(4) Autoattack (AA): Combining multiple attack methods to generate more deceptive adversarial samples in an automated manner;

(5) MIFGSM: On the basis of FGSM, a momentum term is introduced to generate adversarial samples through accelerated gradient descent.

Secondly, in order to verify the effectiveness of the proposed frequency-domain transformation adversarial attack algorithm based on channel awareness, the following attack algorithms based on channel information are selected as comparative experiments in this paper:

(1) Channel inversion attack: The attacker alters the characteristics of the wireless communication channel (such as channel gain, phase, etc.), causing abnormal changes in the channel state;

(2) Maximum disturbance power attack (MRPP): The attacker exploits the phase of the target communication channel obtained.

Adjust the power of the interfering signal in a targeted manner based on relevant information (such as channel status information, noise characteristics, etc.) and the size of the disturbance limited by power is changed to complete the attack.

As shown Table 2, under the CNN1D model and the RadioML2016.10a dataset, the frequency-domain adversarial attack method (FTA) is significantly superior to other attack algorithms in terms of attack efficiency and perturbation concealment. Specifically, the misclassification rate of FTA is 2.3% higher than that of the suboptimal method AA. Meanwhile, its perturbation energy is the lowest among the comparison methods, decreasing by 3.9% and 10.7% respectively compared with PGD and BIM. This result indicates that FTA, through the frequency-domain sparse perturbation injection strategy, can effectively cross the classifier decision boundary under extremely small perturbations.

In terms of confidence offset, the adversarial samples generated by FTA show a high degree of directional misleading. The mean confidence of the error class is close to the theoretical upper limit 1, while the confidence of the correct class is compressed to a value close to zero, which is 85.3% lower than that of FGSM. Although the ACAC and ACTC indicators of FTA are not significantly different from some mature methods (such as MIFGSM), its unique frequency-domain masking mechanism achieves the optimal balance between attack effectiveness and concealment by suppressing the disturbance of redundant frequency bands.

As shown in Table 3, under the experimental framework of the CNN2D model and the RadioML2016.10a dataset, the frequency-domain adversarial attack method (FTA) demonstrates balanced performance advantages. Although its attack success rate is slightly lower than that of the current optimal automatic attack AA, its disturbance concealment is significantly better than AA, reducing by 11.8% and 19.8%, respectively, compared with MIFGSM and FGSM. This result indicates that FTA, through the frequency-domain sparsification perturbation generation strategy, effectively inhibits the diffusion of redundant energy while ensuring the attack effectiveness, achieving an efficient balance between attack intensity and concealment.

Further analysis reveals that the confidence misleading ability of FTA differs from that of AA by only 2.3%, while its cross-model transferability is superior to that of AA, indicating that its perturbation mode has greater generalization potential. It is worth noting that all adversarial samples in the experimental design are derived from clean samples that can be correctly classified by the target model, resulting in a non-uniform distribution of the disturbance-to-signal ratio (PNR). This will cause local inconsistencies in the relationship between the $L_{2}$ norm and PNR, and it is necessary to optimize the data balance through dynamic signal-to-noise ratio sampling in subsequent studies.

**Figure 12:**
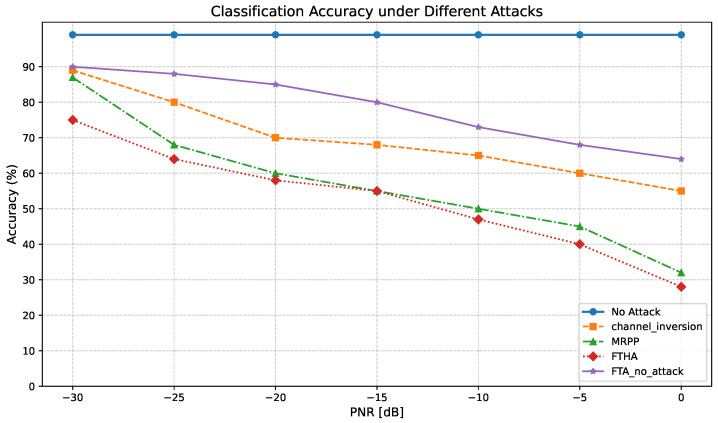
Classification accuracy under different attacks.

Figure 12 shows the relationship between the classifier accuracy and PNR under the proposed target white-box adversarial attack with precise channel information and compares it with the channel inversion attack and the maximum perturbation power attack considering the information. It can be observed that the FTA algorithm without considering the channel effect has very poor performance, close to the no-attack situation in the low PNR region. This is because the wireless channel changes the phase and amplitude of the disturbance perceived by the receiver. Furthermore, compared with the MRPP attack, the target channel inversion attack performs poorly, which indicates the importance of the received perturbation power to the performance of the classifier at the receiver. The classification accuracy of the FTHA proposed in this paper is higher than that of MRPP and the channel inversion attack, indicating that it has better performance within a certain range of perturbation power.

It can be seen from Table 4 and Table 5 that under Gaussian channel conditions, the performance of the FTHA method on the CNN1D and CNN2D models is significantly better than that of other attack methods, especially in terms of the error classification rate and the average confidence of error class prediction. FTHA, as an improved method of adding channel information on the basis of FTA, can better adapt to the Gaussian channel environment and make full use of the channel characteristics to generate more threatening adversarial samples. On the CNN1D model, the misclassification rate of FTHA increased from 68.7% of FTA to 79.90%, while maintaining a relatively high average confidence level of error class prediction (0.935), indicating that it can mislead the model classification more effectively under channel conditions.

On the CNN2D model, FTHA further increased the misclassification rate to 80.50%, while ACAC reached 0.912, significantly higher than other methods, demonstrating its strong attack capability under complex models. In contrast, the performance of other methods such as FGSM and PGD in the Gaussian channel environment is relatively weak, especially in the CNN2D model. The misclassification rate of FGSM is only 28.00%, significantly lower than that of FTHA, which further highlights the advantages of FTHA under channel conditions. Overall, FTHA has demonstrated stronger attack capabilities and robustness in the Gaussian channel environment by combining channel information. Whether on the CNN1D or CNN2D models, it provides a better solution for adversarial sample generation.

It can be seen from the results of Table 6 and Table 7 that the accuracy of channel estimation has a decisive influence on the performance of the FTHA method, which directly reflects the key role of precise channel information in adversarial attacks. In CNN1D and CNN2D models, the performance of FTHA (SDRNet) has always been superior to other methods, especially in terms of the misclassification rate and the average confidence of misclass prediction. This is mainly because SDRNet can estimate the channel state more accurately, thereby generating more targeted adversarial samples. In contrast, FTHA (MMSE) has a significantly weaker attack effect than other methods due to its lower channel estimation accuracy. Especially in the CNN2D model, its MR Is only 77.50%, which is significantly lower than 80.50% of FTHA (SDRNet). This gap indicates that the error of channel estimation will directly affect the generation quality of adversarial samples, resulting in a decline in the attack effect.

## 8. Conclusions

To sum up, this study successfully proposed a new channel estimation method based on deep learning, giving full play to the advantages of deep learning in data processing and feature extraction, and achieving high-precision estimation of sparse channels in OFDM systems. On this basis, further innovation was carried out and a channel-aware adversarial sample generation method based on frequency-domain transformation was proposed. It effectively integrates channel estimation and frequency-domain transformation techniques, significantly improving the generation effect of adversarial samples.

Judging from the phenomena revealed by the research results, deep learning models can capture channel features more accurately. In the complex OFDM system environment, they show stronger adaptability and accuracy compared with traditional algorithms. This theoretical achievement not only enriches the research methods in the field of channel estimation, but also provides new ideas for the optimization of communication systems. In terms of practical significance, high-precision channel estimation and more effective adversarial sample generation methods contribute to enhancing the stability and security of communication systems, providing more reliable technical support for practical communication applications.

Although this study has achieved remarkable results, there is still room for further research. In the future, the performance optimization of deep learning models under different channel conditions can be deeply explored, and studies can be conducted on how to further enhance the universality and concealment of adversarial samples. Meanwhile, studies can apply these methods to more complex communication scenarios, verify their effectiveness and feasibility, and contribute more to the development of communication technology.

## Figures and Tables

**Table 1:** Default parameters of the channel simulation system.

Parameter Name	Parameter Valve
Symbol period	$6.4\times 10^{-6}$
Multipath quantity	3
Sampling period	$1\times 10^{-6}$
Number of loop iterations	100
Delay time	0, $1\times 10^{-6}$, $2\times 10^{-6}$
Root mean square delay spread	$4\times 10^{-6}$

**Table 2:** Performance of different methods on CNN1D under ideal channel conditions.

Attack Method	MR (%)	ACAC	ACTC	$L_{2}$
PGD	81.0	0.990	0.007	1.104
FGSM	31.7	0.835	0.072	1.420
BIM	81.3	0.902	0.007	1.190
AA	91.1	0.044	0.010	1.202
MIFGSM	82.9	0.966	0.005	1.325
FTA	93.4	0.938	0.011	1.062

**Table 3:** Performance of different methods on CNN2D under ideal channel conditions.

Attack Method	MR (%)	ACAC	ACTC	$L_{2}$
PGD	72.20	0.849	0.085	1.247
FGSM	32.40	0.882	0.048	1.579
BIM	71.00	0.848	0.087	1.233
AA	81.01	0.876	0.069	1.332
MIFGSM	71.90	0.844	0.081	1.437
FTA	76.82	0.856	0.071	1.267

**Table 4:** Performance of different methods on CNN1D under gaussian channel conditions.

Attack Method	MR (%)	ACAC	ACTC	$L_{2}$
PGD	65.00	0.950	0.020	1.300
FGSM	25.00	0.800	0.100	1.600
BIM	65.05	0.850	0.020	1.350
AA	69.60	0.800	0.030	1.400
MIFGSM	67.00	0.920	0.015	1.500
FTA	68.70	0.900	0.025	1.250
FTHA	79.90	0.935	0.019	1.268

**Table 5:** Performance of different methods on CNN2D under gaussian channel conditions.

Attack Method	MR (%)	ACAC	ACTC	$L_{2}$
PGD	65.00	0.820	0.090	1.412
FGSM	28.00	0.850	0.060	1.734
BIM	64.00	0.820	0.090	1.425
AA	75.00	0.850	0.070	1.511
MIFGSM	65.50	0.810	0.085	1.669
FTA	67.58	0.830	0.075	1.458
FTHA	80.50	0.912	0.008	1.318

**Table 6:** Performance of FTHA of different channel estimation methods on CNN1D.

Attack Method	MR (%)	ACAC	ACTC	$L_{2}$
FTA (No Channel)	68.70	0.900	0.025	1.250
FTHA (MMSE)	76.70	0.858	0.014	1.200
FTHA (OMP)	78.80	0.935	0.008	1.250
FTHA (LS)	78.50	0.868	0.005	1.300
FTHA (SDRNet)	79.90	0.935	0.005	1.350

**Table 7:** Performance of FTHA of different channel estimation methods on CNN2D.

Attack Method	MR (%)	ACAC	ACTC	$L_{2}$
FTA (No Channel)	67.58	0.830	0.075	1.458
FTHA (MMSE)	77.50	0.831	0.018	1.242
FTHA (OMP)	75.30	0.820	0.011	1.132
FTHA (LS)	78.50	0.843	0.008	1.305
FTHA (SDRNet)	80.50	0.912	0.008	1.318

## Data Availability

The first dataset was random. Relevant data files can be obtained from the corresponding author upon reasonable request.The second dataset used in this study is a publicly available dataset RadiOML 2016.10A, which can be accessed via https://www.deepsig.ai/datasets (accessed on 29 April 2025). Detailed information about the dataset is provided in the Methods section. Experimental results supporting the findings of this study are presented in the manuscript (Tables and Figures). Additional raw data or analysis results can be provided by the authors upon request.
